# TCN-MAML: A TCN-Based Model with Model-Agnostic Meta-Learning for Cross-Subject Human Activity Recognition

**DOI:** 10.3390/s25134216

**Published:** 2025-07-06

**Authors:** Chih-Yang Lin, Chia-Yu Lin, Yu-Tso Liu, Yi-Wei Chen, Hui-Fuang Ng, Timothy K. Shih

**Affiliations:** 1 Department of Mechanical Engineering, National Central University, Taoyuan City 32001, Taiwan; 2 Department of Computer Science and Information Engineering, National Central University, Taoyuan City 32001, Taiwanneoliu@g.ncu.edu.tw (Y.-T.L.); yiwei.chen@g.ncu.edu.tw (Y.-W.C.); 3 Department of Computer Science, Universiti Tunku Abdul Rahman, Kampar 31900, Perak, Malaysia; nghf@utar.edu.my

**Keywords:** human activity recognition, MAML, TCN, wireless sensor networks

## Abstract

Human activity recognition (HAR) using Wi-Fi-based sensing has emerged as a powerful, non-intrusive solution for monitoring human behavior in smart environments. Unlike wearable sensor systems that require user compliance, Wi-Fi channel state information (CSI) enables device-free recognition by capturing variations in signal propagation caused by human motion. This makes Wi-Fi sensing highly attractive for ambient healthcare, security, and elderly care applications. However, real-world deployment faces two major challenges: (1) significant cross-subject signal variability due to physical and behavioral differences among individuals, and (2) limited labeled data, which restricts model generalization. To address these sensor-related challenges, we propose TCN-MAML, a novel framework that integrates temporal convolutional networks (TCN) with model-agnostic meta-learning (MAML) for efficient cross-subject adaptation in data-scarce conditions. We evaluate our approach on a public Wi-Fi CSI dataset using a strict cross-subject protocol, where training and testing subjects do not overlap. The proposed TCN-MAML achieves 99.6% accuracy, demonstrating superior generalization and efficiency over baseline methods. Experimental results confirm the framework’s suitability for low-power, real-time HAR systems embedded in IoT sensor networks.

## 1. Introduction

The field of human activity recognition (HAR) has garnered increasing attention, emerging as a dynamic area of research, particularly in the realm of device-free recognition. Traditional HAR methods typically rely on camera-based and sensor-based systems. However, these methods often incur significant costs and require wearable devices, which can lead to user discomfort and inconvenience [1,2,3,4,5]. A notable advancement in device-free recognition is the utilization of Channel State Information (CSI) derived from Wi-Fi signals [6], a method that has gained significant traction in recent studies [7,8,9,10].

**Figure 1:**
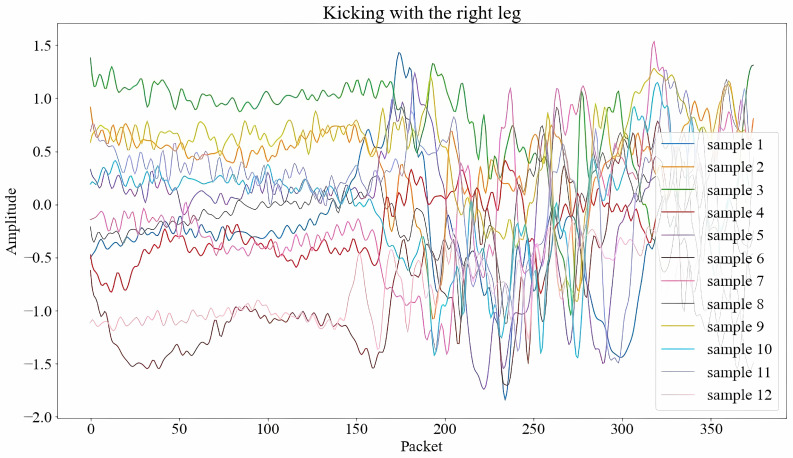
Significant variations in Wi-Fi CSI signals of the same “Kicking with the right leg” activity among different samples from the HHI dataset [7].

HAR systems based on Wi-Fi are particularly notable for their wide range of applications in fields such as healthcare, security, and elderly care [11]. These systems leverage Wi-Fi signals to detect and analyze human activities within indoor settings, offering a non-intrusive and user-friendly alternative to traditional sensor- and device-based approaches. Despite their numerous benefits, Wi-Fi-based HAR systems face several challenges that hinder their widespread adoption. A primary challenge is the inherent variability of Wi-Fi signals, which can fluctuate across different timeframes, environments, and individuals. This variability, stemming from factors such as signal interference, environmental changes, and individual physical characteristics, creates significant obstacles to achieving accurate and consistent activity recognition. The impact of these signal fluctuations is demonstrated in Figure 1, where Wi-Fi CSI signals for the ’kicking with the right leg’ action from the Human-to-Human Interaction (HHI) dataset [7] exhibit marked differences between various subject pairs. These variations, stemming from inter-subject differences and temporal changes, pose considerable challenges to consistent cross-subject recognition, even for human observers.

Traditional machine learning methods often struggle with limited training data, particularly in cross-subject recognition tasks. To address this challenge, few-shot learning [12] has emerged as a promising solution. This paper introduces a novel Wi-Fi-based HAR framework designed to mitigate issues arising from cross-subject signal variability and data scarcity. Our approach integrates Temporal Convolutional Networks (TCNs) [13] with Model-Agnostic Meta-Learning (MAML) [12], forming the TCN-MAML framework, which facilitates rapid adaptation to new subjects with minimal fine-tuning. Compared to recurrent models such as LSTM and attention-based models like Transformer, TCNs offer a lightweight architecture with lower memory and computational requirements, rendering them highly suitable for real-time deployment in resource-constrained environments. Additionally, MAML optimizes model initialization, significantly reducing training overhead while ensuring fast convergence.

To assess the scalability and deployment feasibility of TCN-MAML, we conducted extensive experiments on the HHI dataset [7], partitioning it into distinct training and testing subsets for evaluation. The use of dilated causal convolutions enhances inference efficiency, thereby enabling fast execution on low-power devices. Furthermore, the ability to adapt with limited labeled data significantly reduces the need for frequent retraining, which is a crucial factor for practical IoT and embedded system deployments. These findings highlight the potential of TCN-MAML in real-world HAR applications, where power efficiency, low latency, and robustness to new subjects are critical considerations. The major contributions of this study are as follows:

- We proposed a novel TCN-MAML model for effective few-shot cross-subject human activity recognition using Wi-Fi signals. To the best of our knowledge, this study is the first to explore the unique integration of Temporal Convolutional Networks with the MAML algorithm for HAR, which surpasses state-of-the-art methods in performance.
- Three augmentation methods specifically designed for Wi-Fi signals were proposed. These methods incorporate variations into the original signals to effectively expand the dataset and enrich its diversity. The application of these proposed augmentation techniques resulted in over 10% improvement in test accuracy compared to the baseline model trained without any augmentation.
- This study is the first to conduct realistic cross-subject experiments by partitioning the HHI dataset into two non-overlapping subsets, where the model was trained on one subset and tested on the other. The proposed approach yields a remarkable recognition accuracy of 99.60% on new subjects.

The remainder of this paper is structured as follows: Section 2 provides a review of related Wi-Fi-based HAR, with a focus on few-shot learning approaches. Section 3 defines the targeted problem and presents a detailed description of the proposed method. Section 4 presents the experiments and results. Lastly, Section 5 concludes the paper and outlines potential avenues for future research.

## 2. Related Work

In recent years, Wi-Fi-based human activity recognition (HAR) has garnered significant attention due to its wide applications in healthcare monitoring, security, elderly care [11], and other domains. While many existing approaches have achieved impressive performance on benchmark datasets, a large portion of prior research tends to overlook or underemphasize the challenge of cross-subject generalization, a critical factor for real-world deployment where models must adapt to new users without retraining. In this section, we review existing Wi-Fi-based HAR methods and several recent HAR systems that employ few-shot learning for cross-domain or cross-subject recognition.

### 2.1. Wi-Fi-Based Human Activity Recognition (HAR)

Early-stage Wi-Fi-based HAR studies primarily relied on Convolutional Neural Network (CNN)-based models. This choice was motivated by the unique activity patterns discernible within Channel State Information (CSI) signals, rendering CNNs an effective option for activity recognition. For instance, Kabir et al. [14] introduced the CSI-based Inception Attention Network (CSI-IANet) for Wi-Fi-based HAR. This model combined CNNs with spatial attention mechanisms and was evaluated using the HHI dataset [7]. Notably, the CSI-IANet achieved an average accuracy of 91.30%, making it the first CNN-based model to surpass the 90% accuracy threshold on this dataset. Subsequently, Shafiqul et al. [6] introduced the HHI-AttentionNet, which incorporated a depth-wise CNN and a customized attention mechanism. Their method utilized a Butterworth low-pass filter to eliminate outliers and high-frequency noise, and a Gaussian smoothing function to mitigate short peaks, achieving an impressive 95.47% accuracy on the HHI dataset. These models achieved strong performance on benchmark datasets but were primarily evaluated in single-subject or controlled cross-subject settings, thereby limiting their ability to generalize to new individuals. One notable instance is H2HI-Net [15], a unified model that combines a Residual Neural Network with a Bi-directional Gated Recurrent Unit (Bi-GRU). The Residual Neural Network encodes spatial embeddings, while the Bi-GRU focuses on temporal embeddings. The spatial and temporal representations are then concatenated with a two-layer DenseNet. The model achieves an average accuracy of 96.39% on the HHI dataset. In our previous work [10], we proposed the TCN-AA model that incorporates data augmentation, an attention mechanism, and Temporal Convolutional Networks (TCNs) to effectively extract features from CSI signals while maintaining a low number of model parameters compared to other methods. TCN-AA achieved an accuracy of 99.42% on the HHI dataset, outperforming H2HI-Net by 3%. However, while TCN-AA enhances feature extraction, it does not explicitly address cross-subject adaptation, thereby rendering it less effective in real-world settings where new individuals are introduced.

In contrast, the proposed TCN-MAML model extends beyond TCN-AA by integrating Model-Agnostic Meta-Learning (MAML), which allows the model to rapidly adapt to new subjects with minimal training samples. Unlike H2HI-Net, which relies on recurrent components, TCN-MAML leverages a fully convolutional structure, rendering it more computationally efficient while achieving strong cross-subject generalization. Our approach not only maintains high accuracy (99.6%) but also demonstrates improved adaptability in scenarios where training data is limited, making it more suitable for real-world deployments.

### 2.2. Few-Shot Learning-Based HAR Approaches

Few-shot learning techniques have been applied in Wi-Fi-based HAR to improve cross-subject generalization, allowing models to recognize activities performed by previously unseen individuals. As shown in Table 1, different few-shot approaches vary in their adaptability, reliance on additional data, and computational feasibility. ProtoNet [16] addresses cross-subject variability by learning distance-based embeddings, but these embeddings remain fixed after training, making it difficult for the model to adapt to highly dynamic CSI signals from new subjects. TOSS [17] enhances cross-domain generalization by integrating semi-supervised meta-learning, where the model is exposed to both labeled and unlabeled target-domain data. While this improves adaptation, it still requires additional labeled target data, which may not always be available in real-world settings. CSI-GDAM [18] applies a graph-based attention mechanism to model relationships between different activities, improving representation learning. However, it requires manually defining graph structures, such as node representations for activity classes and edge weights based on inter-class relationships, which constitutes extensive feature engineering. This manual design process limits the model’s flexibility and scalability across different environments. CAUTION [19], designed specifically for Wi-Fi-based authentication, transforms CSI signals into a low-dimensional feature space for identity verification rather than general HAR. While effective in authentication tasks, its reliance on manual feature extraction further restricts its adaptability. In contrast, our TCN-MAML model eliminates the need for predefined embeddings and additional labeled target data while maintaining high adaptability to new subjects. Unlike CSI-GDAM and CAUTION, it does not require feature engineering and is applicable to general HAR applications. By integrating TCN’s efficient temporal feature extraction with MAML’s gradient-based adaptation, TCN-MAML allows for fast generalization with minimal data, reducing the need for frequent retraining. This makes it a scalable, data-efficient, and computationally feasible solution for real-world Wi-Fi-based HAR applications, such as healthcare monitoring, security surveillance, and smart home automation.

### 2.3. Recent Advances in Deep Learning-Based Wi-Fi HAR

Recent studies have continued to improve Wi-Fi-based human activity recognition (HAR) by leveraging deep learning models and signal selection strategies. Sousa et al. [20] proposed a CNN-based subcarrier selection network that automatically identifies the most discriminative CSI subcarriers, significantly reducing input complexity while maintaining high recognition accuracy. This approach jointly performs subcarrier selection and classification, offering a lightweight alternative for real-world deployment. In another study, Shahverdi et al. [21] explored the use of various deep learning architectures, including CNNs, LSTMs, and GRUs, to model temporal patterns in CSI signals. Their experiments demonstrated that GRU models were particularly effective in capturing time-dependent activity features, leading to robust HAR performance. These works reflect an ongoing trend toward optimizing both input representation and model efficiency in recent CSI-based HAR research.

## 3. Proposed Method

In this section, we first detail the CSI dataset used and define the problem addressed in this study. Next, we present the proposed pre-processing and augmentation techniques. Finally, a detailed discussion of the novel Temporal Convolutional Network with Model-Agnostic Meta-Learning (TCN-MAML) model is provided.

### 3.1. Preliminaries

#### 3.1.1. The HHI CSI Dataset

The publicly accessible Human-to-Human Interaction (HHI) CSI dataset [7] is employed in this study. This dataset consists of CSI data collected from 66 individuals, comprising 40 unique subject pairs, each instructed to execute 12 distinct interaction activities ($N_{c}$ = 12). These activities include actions such as approaching, departing, handshaking, high-fiving, hugging, kicking with the left leg, kicking with the right leg, pointing with either hand, and various punching and pushing motions. Each subject pair performed 10 trials for each activity, culminating in a total of 4800 samples. Each trial lasted between 5 and 6 s. In every trial, the subjects were asked to stand still for 2 s and then permitted to start the interaction, resulting in packet lengths ranging from 1040 to 2249 ($N_{p}$ = [1040, 2249]). The data-collection environment was set up using a 2 × 3 Multiple-Input Multiple-Output (MIMO) system, comprising 2 transmitters ($N_{t}$ = 2) and 3 receivers ($N_{r}$ = 3). The Channel State Information (CSI) data were obtained using a publicly available CSI tool [22] across 30 subcarriers ($N_{s}$ = 30). Readers may refer to [7] for additional information regarding the data-collection process.

#### 3.1.2. Problem Definition

**Figure 2:**
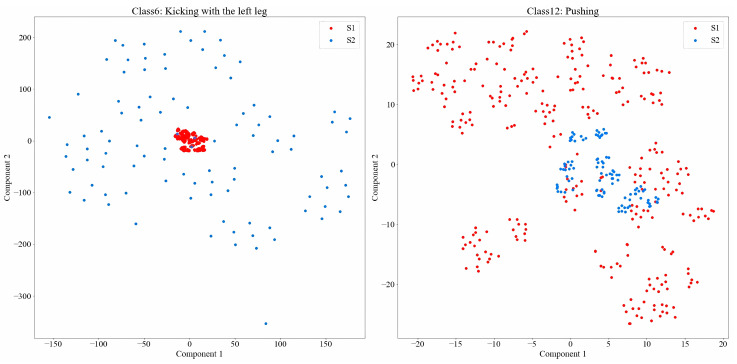
Visualization of CSI data using t-SNE of similar activities from $D^{s_{1}}$ and $D^{s_{2}}$. Red dots correspond to data from $D^{s_{1}}$ and blue dots represent data from $D^{s_{2}}$.

In this study, we partition the HHI dataset based on subject pairs to rigorously evaluate cross-subject generalization. Let D denote the HHI dataset, and S represent all subject pairs within it. The dataset is divided into two distinct subsets, $S_{1}$ and $S_{2}$, where $S_{1}$ and $S_{2}$, and where $S_{1}\cup S_{2}=S$ and $S_{1}\cap S_{2}=\emptyset$. This ensures that no subject pairs overlap between training and testing. Specifically, $S_{1}$ contains 30 subject pairs for training, while $S_{2}$ consists of 20 subject pairs for testing. The number of activity types remains identical across both subsets, i.e., $N_{c}^{S_{1}}=N_{c}^{S_{2}}=N_{c}$. The Wi-Fi CSI samples collected by $S_{1}$ and $S_{2}$ are denoted as $D^{s_{1}}$ (source domain) and $D^{s_{2}}$ (target domain), respectively. Our objective is to develop a Wi-Fi-based HAR model that is trained on $D^{s_{1}}$ and can effectively generalize to recognize the same activities in $D^{s_{2}}$. To demonstrate the challenge in Wi-Fi-based cross-subject recognition, Figure 2 visualizes CSI data using t-Distributed Stochastic Neighbor Embedding (t-SNE) for similar activities from $D^{s_{1}}$ and $D^{s_{2}}$. It is evident that significant distribution disparities exist between $D^{s_{1}}$ and $D^{s_{2}}$, rendering cross-subject recognition a highly challenging task.

We acknowledge that partitioning by subject pairs may introduce potential biases, as different individuals exhibit variations in physical characteristics, movement patterns, and signal interactions. However, this setup reflects real-world deployment scenarios, where the model must recognize activities performed by previously unseen individuals rather than memorizing subject-specific patterns. Despite these challenges, our experimental results demonstrate that the proposed model achieves high recognition accuracy under this strict evaluation setup, validating its robustness in handling cross-subject variability. A detailed discussion on the impact of this partitioning strategy on generalization performance will be presented in Section 4.

### 3.2. Preprocessing

**Figure 3:**
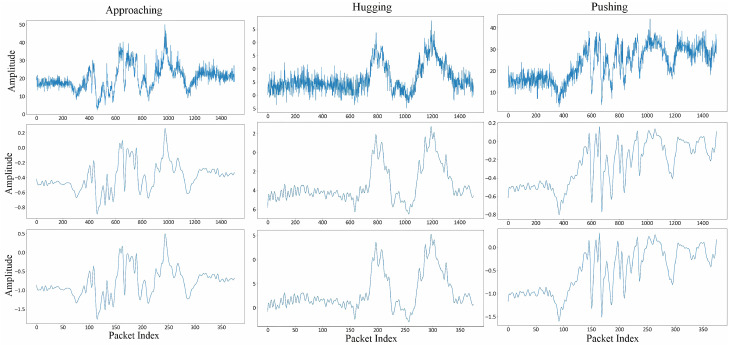
The sample preprocessing results of amplitude data for three interactions. The first row is the raw data; the second row is the data after normalization and Butterworth low-pass filtering; and the third row is the data after 1D-DWT down-sampling.

Direct use of raw CSI values for HAR is challenging due to the inherent noise within the CSI signals. Therefore, preprocessing CSI values is crucial before further analysis. While the transmitter sends data at a predetermined frequency during the collection phase, the actual sampling rate can fluctuate due to various internal and external factors. In the HHI dataset, packet sizes range from 1040 to 2249. To safeguard data quality, a threshold of 1500 packets ($N_{p}$ = 1500) is established, and data with fewer packets than this threshold is discarded. To maintain consistency and streamline subsequent analysis, packet counts exceeding 1500 are normalized to this value. This procedure guarantees that all input data adheres to a uniform size, represented as ($N_{t}$*$N_{r}$, $N_{p}$, $N_{s}$). The values of each Transmitter–Receiver pair (TR pair) are then standardized to a range of [−1, 1] to ensure that the CSI values are comparable across different TR pairs. To further improve data quality, a low-pass Butterworth filter is applied to each TR pair. This filter is particularly effective at eliminating high-frequency noise that could potentially distort the CSI signals. Lastly, a one-dimensional Discrete Wavelet Transform (1D-DWT) is applied twice to down-sample the processed signal. The original signal is decomposed into approximation coefficients, which retain low-frequency components, and detailed coefficients, which contain high-frequency noise. Since the most relevant activity information resides in the low-frequency domain, we retain the approximation coefficients while reducing the packet length from 1500 to 375, effectively lowering computational complexity while preserving key motion features. Compared to alternative techniques, 1D-DWT offers superior temporal structure preservation. Unlike Short-Time Fourier Transform (STFT), which suffers from fixed window-size trade-offs, or Principal Component Analysis (PCA), which may remove essential nonlinear dependencies, 1D-DWT dynamically adjusts to signal variations while achieving effective compression. As shown in Figure 3, the application of a Butterworth filter removes high-frequency noise, while repeating 1D-DWT maintains key feature patterns, thereby reducing both data storage requirements and training time, and making it ideal for resource-constrained environments.

### 3.3. Data Augmentation

While an abundance of large image datasets, such as ImageNet [23] and COCO [24], exists for image recognition tasks, no such large datasets are available for Wi-Fi-based applications. Consequently, the application of data augmentation techniques becomes crucial for Wi-Fi datasets and for training Wi-Fi-based HAR models. This study proposes three augmentation techniques tailored for Wi-Fi data to mitigate the data scarcity problem.

#### 3.3.1. Random Dropout

Random dropout is a data augmentation mechanism where a fraction of the CSI values is randomly set to zero to simulate unstable Wi-Fi transmission environments (i.e., packet loss). The probability of setting a value to zero, denoted as λ, is randomly selected from the range (0, 0.07). This augmentation technique was inspired by the success of dropout regularization in deep learning. Hence, it is reasonable to expect that applying dropout as an augmentation technique for Wi-Fi data would yield positive results.

#### 3.3.2. Inter-Class Mixing

CSI signals are susceptible to external interferences such as background radio noise and disturbances caused by moving subjects, causing the received signals to fluctuate over time. To improve the resilience of the HAR model to noisy signals, an augmentation technique called inter-class mixing is proposed. This technique involves combining samples from different action classes to enhance the diversity of the dataset. The mixing procedure is represented by the following equation:

(1) $$D=A\times \left(1-\varepsilon_{1}\right)+B\times \varepsilon_{2}+C\times \varepsilon_{3}$$

A new sample, *D* is generated by combining three existing samples, A, B, and C. Sample D inherits its class label from sample A, whereas samples B and C have different labels from sample A. Throughout our experiments, the value of $\varepsilon_{k}$ (where k∈{1,2,3}) was randomly chosen from the range (0, 0.05). This mixing technique helps introduce diversity into the dataset and thereby enhances the model’s robustness.

#### 3.3.3. Intra-Class Mixing

It is observed that when different subjects perform the same activity, significant variations occur in the received CSI signals, caused by discrepancies in subjects’ heights and body shapes. The disturbance from subject movement is more profound than background noise. Motivated by this observation, the intra-class mixing augmentation technique is proposed. Intra-class mixing utilizes the same mixing equation as in Equation (1), with the distinction that A, B, and C are samples from the same action class. The intra-class mixing technique should be beneficial in strengthening the model’s resilience to intra-class variations.

### 3.4. TCN-MAML Model

The proposed model parameters are listed in Table 2.

#### 3.4.1. TCN Model

**Figure 4:**
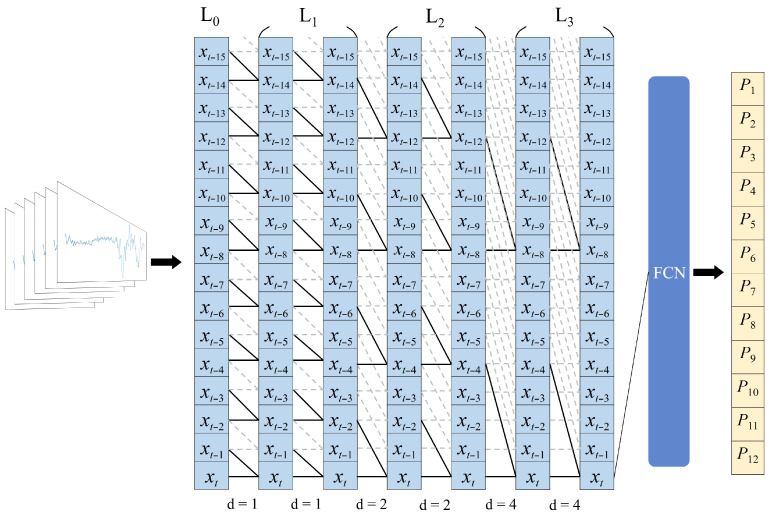
An overview of the architecture of the TCN model used in this paper. A kernel size of two is shown for illustration purposes.

**Figure 5:**
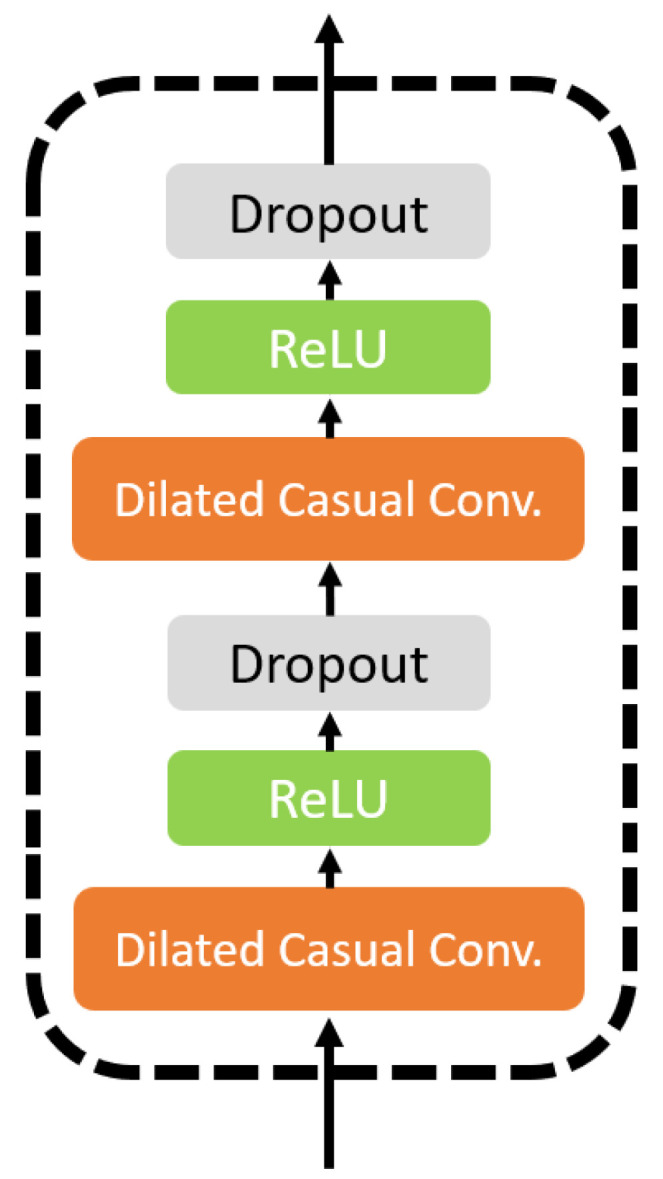
The details of a TCN layer.

CSI signals, by nature, are time-series data. As such, sequential deep learning models such as Long Short-Term Memory (LSTM) [25] and Transformer [26] are traditionally well-suited for processing such signals. However, these models demand significant computational resources, require large datasets, and often exhibit long training times, rendering them impractical for real-time Wi-Fi-based human activity recognition (HAR) applications. LSTMs process data sequentially, leading to high memory consumption and limited parallelization, while Transformers rely on self-attention mechanisms with quadratic complexity, making them computationally expensive. In contrast, Temporal Convolutional Networks (TCNs) [13] offer a more efficient alternative by employing one-dimensional causal and dilated convolutions, which effectively capture long-range temporal dependencies without the need for recurrence mechanisms. Compared to LSTMs and Transformers, TCNs reduce training time, enable parallel computation, and maintain high recognition accuracy with fewer computational resources. An overview of the TCN architecture used in this study is shown in Figure 4. Each TCN layer begins with a dilated causal convolution layer, followed by a ReLU activation layer and a dropout layer, as shown in Figure 5.

#### 3.4.2. TCN with MAML

To facilitate cross-subject recognition, the TCN model must efficiently adapt to new subject pairs. To achieve this, we propose TCN-MAML, which integrates Temporal Convolutional Networks (TCNs) with Model-Agnostic Meta-Learning (MAML) [12]. Unlike traditional training, which requires extensive retraining for each new subject, MAML learns an optimal model initialization, allowing for rapid adaptation with minimal labeled samples. This is particularly advantageous for Wi-Fi CSI-based HAR, where collecting large labeled datasets for every new user is impractical due to inter-subject variability.

MAML’s gradient-based adaptation allows TCN-MAML to adjust directly from task-specific gradients, rather than relying on predefined embeddings or handcrafted features. This enables the model to automatically generalize to new subjects, significantly improving cross-subject recognition while maintaining computational efficiency. Unlike approaches that require explicit task-specific engineering, TCN-MAML autonomously learns transferable representations, making it well-suited for deployment in low-resource environments. By combining TCN’s efficient feature extraction with MAML’s adaptability, TCN-MAML ensures both accuracy and scalability in real-world Wi-Fi HAR applications, including healthcare monitoring and security surveillance, where minimizing retraining efforts is crucial for practical deployment.

The MAML algorithm achieves this by dividing model training into two phases: inner-loop optimization and outer-loop optimization. For each meta-learning episode *i*, an *N*-way *K*-shot task $T_{i}$ = $\left[S_{i},Q_{i}\right]$ is randomly sampled from the training dataset. The model (e.g., TCN), denoted as $f_{\theta_{i}}$ with model weight $\theta_{i}$ at the *i*th episode, together with the support set $S_{i}$, is used to kick-start the inner-loop optimization process. At the starting of the inner-loop optimization, a temporary weight $\theta_{t}mp$ is created and initialized to $\theta_{i}$. The temporary model $f_{\theta_{t}mp}$ is then fine-tuned on $S_{i}$ with a few gradient descents. Next, the model with updated $\theta_{t}mp$ is evaluated on the query set $\theta_{i}$ to produce the meta-training loss. The meta loss is then backpropagated in the outer-loop optimization process to update $\theta_{i}$. This process is repeated until the model has been trained on all episodes. Note that at each training episode, the N classes are randomly selected so that they might be different between episodes. The inner-loop optimization and outer-loop optimization can be formulated as:

Inner Loop:

(2) $$\theta_{tmp}^{\left(m\right)}=\left\{\begin{array}{cc} \theta_{i}-\alpha \times \nabla_{\theta }L_{T_{i}}\left(S_{i},f_{\theta_{i}},Y_{S_{i}}\right), & \mathrm{if}\;m=1\\ \theta_{tmp}^{\left(m-1\right)}-\alpha \times \nabla_{\theta }L_{T_{i}}\left(S_{i},f_{\theta_{tmp}^{\left(m-1\right)}},Y_{S_{i}}\right), & \mathrm{if}\;1<m\leq M \end{array}\right.$$

Outer Loop:

(3) $$\theta_{i+1}=\theta_{i}-\beta \times \nabla_{\theta }L_{T_{i}}\left(Q_{i},f_{\theta_{tmp}^{\left(M\right)}},Y_{Q_{i}}\right),\;Q_{i}\in D^{s_{1}}\cup D^{s_{2}}$$

where:

- $\theta_{i}$: model parameters before task *i* adaptation;
- $\theta_{tmp}^{\left(m\right)}$: task-specific parameters after *m* inner-loop updates;
- α,β: learning rates for inner- and outer-loop updates, respectively;
- $S_{i}$, $Q_{i}$: support and query sets sampled from task $T_{i}$;
- $Y_{S_{i}}$, $Y_{Q_{i}}$: ground truth labels for the support and query sets;
- $f_{\theta }$: model parameterized by θ;
- $L_{T_{i}}$: task-specific loss function;
- *M*: number of inner-loop updates.

where $S_{i}$ and $Q_{i}$ are the sampled support set and query set, respectively. M denotes the total number of inner-loop update iterations, and m denotes the mth update iteration. α and β are the learning rates, and $Y_{S_{i}}$ and $Y_{Q_{i}}$ represent the ground truth for the support set and query set, respectively. After MAML training, the final model should be able to adapt to a new task through a few fine-tuning steps using a small support set sampled from the new domain.

In the proposed TCN-MAML model, the support sets used in the inner-loop optimization are sampled from the $D^{s_{1}}$ subset ($S_{i}\in D^{s_{1}}$). However, the query sets in the outer-loop optimization are sampled from both $D^{s_{1}}$ and $D^{s_{2}}$ ($Q_{i}\in \;D^{s_{1}},D^{s_{2}}$). Note that the support set and the query set contain samples from the same N classes, even though the samples might come from different subsets. By including samples from both the source and target domains in the outer-loop optimization process, the TCN model’s adaptability to the target domain is effectively enhanced, thereby enabling cross-domain recognition. The proposed TCN-MAML model requires gathering a few labeled samples from the target domain to perform fine-tuning for that domain. In many real-world situations, it is reasonable to assume that obtaining samples from the target domain is relatively straightforward [17]. For instance, when deploying smart devices in a new environment, it is often customary to request that users engage in specific tasks or activities to facilitate the calibration of these devices for the new environment.

## 4. Experiments

In this section, extensive experiments are carried out to demonstrate the effectiveness of the TCN-MAML model in cross-subject human-to-human interaction recognition.

### 4.1. Experimental Setup

For the *N*-way *K*-shot few-shot learning setup, the value of *N* is set to 5 and *K* is set to 1. As such, in each training episode, the support set S contains 5×1=5 samples randomly sampled from the $D^{s_{1}}$ subset of the HHI dataset. The size of the query set Q is set to 1, and the query samples are randomly sampled from both $D^{s_{1}}$ and $D^{s_{2}}$ subsets of the HHI dataset (see Section 3 for the dataset split). To ensure adequate query samples are drawn from both subsets, we set the sampling ratio to 3:1 for $D^{s_{1}}$ and $D^{s_{2}}$, respectively.

The TCN-MAML model is trained over 30 meta-epochs, with each epoch comprising multiple 5-way 1-shot tasks. The total training time is approximately 6 h. All experiments were conducted on a computer equipped with a GeForce RTX 1060 graphics card (NVIDIA, Santa Clara, CA, USA) and running on the Windows 10 operating system with Python version 3.8 and the PyTorch 1.10.1 deep learning framework. The model is trained using the AdamW optimizer with an exponential learning rate decay of 0.988 per epoch.

### 4.2. Performance Evaluation

#### Cross-Subject Recognition Performance

**Figure 6:**
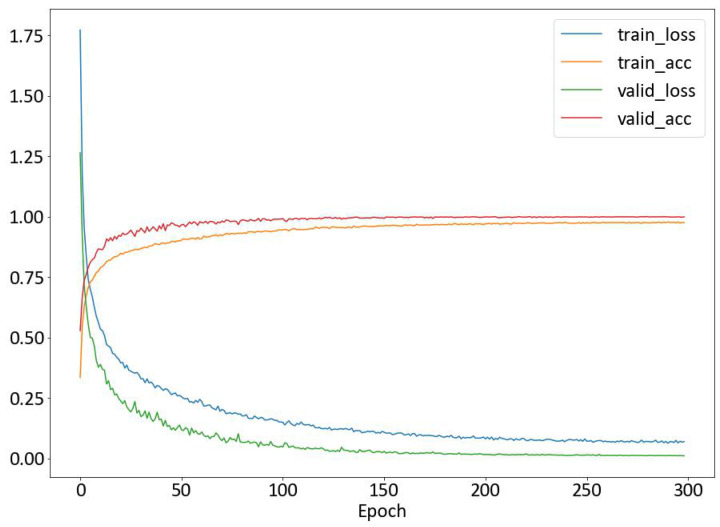
The accuracy and loss curves of the TCN-AA model trained and validated on $D^{s_{1}}$.

**Figure 7:**
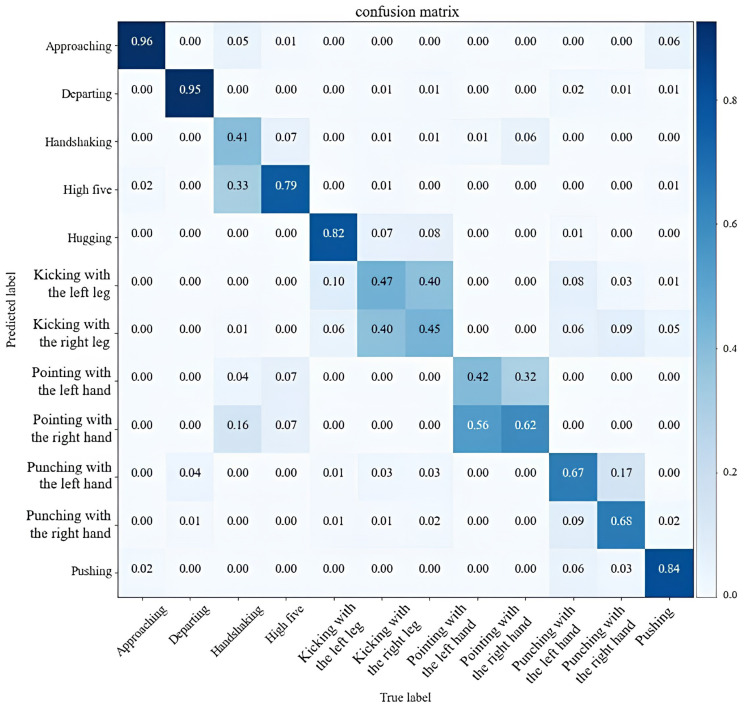
The confusion matrix of the TCN-AA model trained on $D^{s_{1}}$ and tested on $D^{s_{2}}$.

The performance of the proposed TCN-MAML model on Wi-Fi-based human activity recognition is evaluated using the HHI dataset. Its performance is compared with our previously proposed TCN-AA model [10], which has been shown to achieve state-of-the-art recognition accuracy on the HHI dataset. To illustrate the efficacy of the models on cross-subject recognition, they are evaluated using two non-overlapping subsets of the HHI dataset. Specifically, both TCN-AA and TCN-MAML models are trained on the $D^{s_{1}}$ subset and evaluated on a separate subset $D^{s_{2}}$. To ensure a fair comparison, both models adhere to the same TCN architecture and employ the same augmentation techniques as described in Section 3. The TCN-AA model is trained using conventional supervised learning techniques, while the TCN-MAML model undergoes 5-way 1-shot learning with MAML. Another difference is that the dropout rate for TCN-AA is configured at 0.5, whereas for TCN-MAML, a dropout rate of 0.1 is chosen to achieve optimal performance. As shown in Figure 6, the TCN-AA model obtain the best overall training accuracy of 97.84% and achieving 99.6% accuracy on the validation set. For more data-processing details, please refer to our previous work in [10]. However, when assessing TCN-AA on the $D^{s_{2}}$ subset that was not included in the training process, its performance drops significantly. As illustrated in the confusion matrix shown in Figure 7, the average recognition accuracy across the 12 different action classes is 67.4%, much lower than the 99.6% validation accuracy on $D^{s_{1}}$.

**Figure 8:**
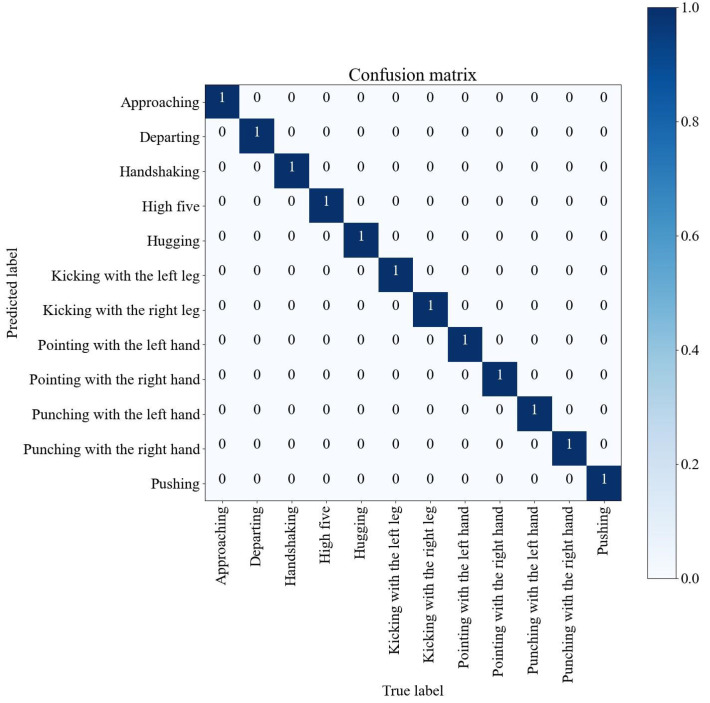
The confusion matrix of the TCN-MAML model trained on $D^{s_{1}}$ and tested on $D^{s_{2}}$.

Furthermore, a notable confusion of the model in recognizing similar activities is observed, such as ’pointing with the left hand’ versus ’pointing with the right hand’ and between ’kicking with the right leg’ and ’kicking with the left leg.’ These results indicate that existing Wi-Fi-based HAR models are not robust enough for cross-subject recognition applications. In contrast, the confusion matrix of TCN-MAML in Figure 8 exhibits greater robustness compared to TCN-AA. The results show that the proposed TCN-MAML model performs significantly better under the same conditions and does not exhibit the issues mentioned above.

To provide a more comprehensive evaluation, we also compare the performance of our proposed method across three different datasets: NTU-Fi HAR [27], UT-HAR [28], and our original HHI dataset. As shown in Table 3, our model achieves a testing accuracy of 99.12% on NTU-Fi HAR and 98.66% on UT-HAR, while maintaining a superior accuracy of 99.6% on the HHI dataset. These results confirm the effectiveness and generalizability of our method across diverse Wi-Fi-based human activity recognition datasets.

To further strengthen the comparative analysis, we provide a benchmark evaluation of our earlier TCN-AA model on the HHI dataset, alongside several recent and representative human activity recognition methods. These include classical models such as SVM, deep learning-based CNN and RNN variants, and attention-enhanced architectures. As presented in Table 4, the TCN-AA model achieves the highest recognition accuracy of 99.42%, outperforming H2HI-Net (96.39%) and HHI-AttentionNet (95.47%).

These results highlight the strong discriminative capability of the TCN-based model on the HHI dataset. The consistent performance improvement across a range of baselines—including CNNs, GRUs, and attention-based frameworks—demonstrates the robustness of our approach. This comparison also serves as an empirical foundation to emphasize the benefits of our full TCN-MAML model, which further enhances cross-subject generalization in data-scarce conditions.

Additionally, to evaluate the actual effect of the augmented data, in the second experiment, the augmented dataset with 800 samples per class was randomly down-sampled to 400 samples per class, making its sample size consistent with the raw dataset. Table 5 shows the classification results using the down-sampled datasets. For this experiment, the testing accuracy increments of each augmentation method are 1.6%, 3%, and 0.6%, respectively. Although the improvement may not be as substantial as when utilizing all augmented samples (Table 6), the augmented samples do enhance the overall diversity and quality of the training data, as evidenced by the observed reduction in loss across all three methods. Note that the validation accuracies in Table 5 and Table 6 are lower than the respective training and testing accuracies. This is mainly due to the much smaller sample sizes in the validation sets since 10-fold cross-validation was applied throughout the experiments.

Furthermore, we evaluated the computational efficiency and memory requirements of our model to assess its feasibility for real-world deployment. The inference speed of TCN-MAML was tested on a dataset containing approximately 4.44 million packets captured over 74 min at 1000 packets per second. The entire evaluation process was completed in less than six minutes, operating at a speed 12 times faster than real-time, making it suitable for real-time applications. In terms of memory usage, the model consumes about 3.15 MB per second, ensuring feasibility for deployment on resource-constrained edge devices. Compared to recurrent architectures such as LSTM and Transformer, which have higher computational demands due to sequential dependencies, the TCN-based model significantly reduces inference time and memory overhead. This efficiency makes TCN-MAML well-suited for practical applications such as healthcare monitoring and security surveillance, where low latency and lightweight deployment are crucial.

## 5. Conclusions

In this study, we introduced TCN-MAML, a novel model for human-to-human activity recognition using Wi-Fi CSI signals. By integrating Temporal Convolutional Networks (TCNs) with Model-Agnostic Meta-Learning (MAML), the proposed framework enabled the recognition of activities performed by previously unseen subject pairs without requiring full retraining. This capability is critical for real-world applications such as patient monitoring, where rapid adaptability and generalization are essential.

**Figure 9:**
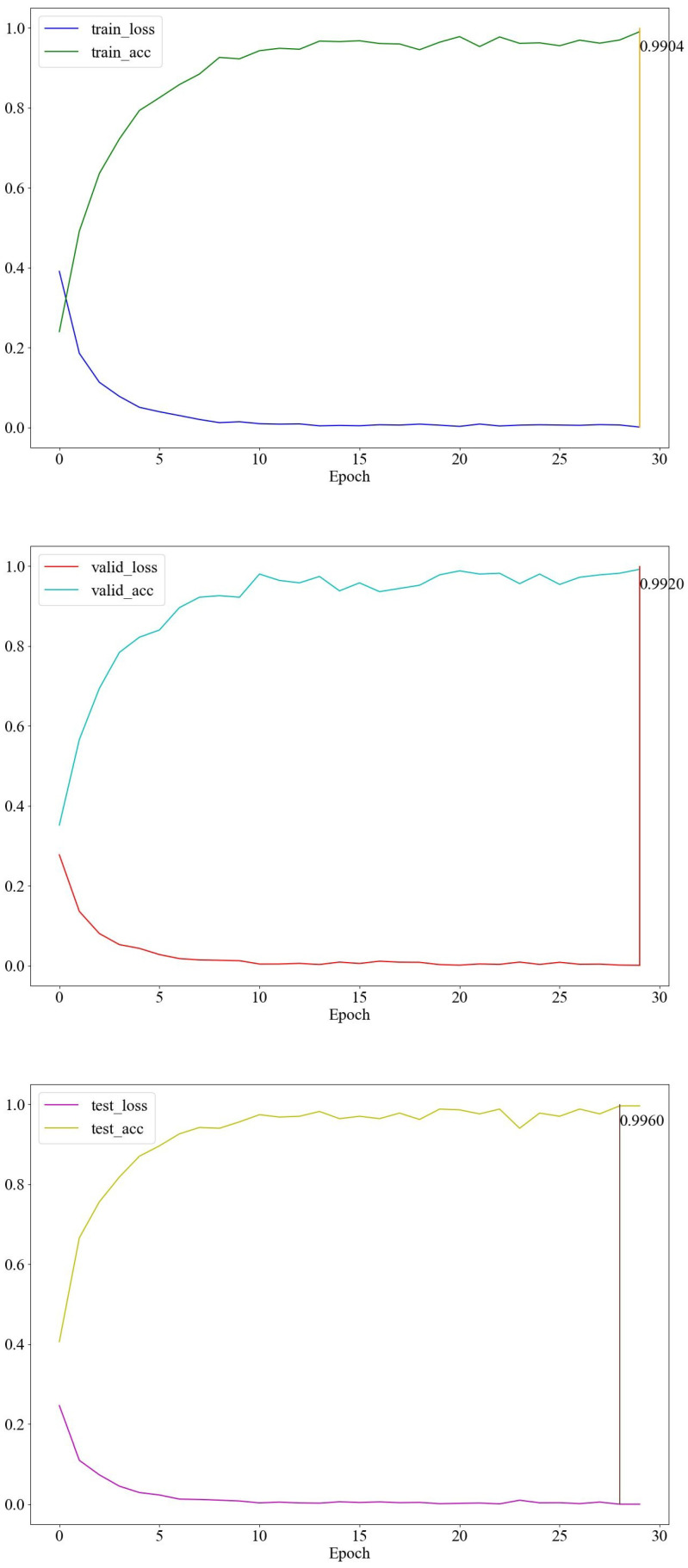
The accuracy and loss curves of the proposed TCN-MAML model.

The model achieved a remarkable accuracy of 99.6% (Figure 9) in the HHI dataset, demonstrating strong generalization between subjects under a challenging benchmark. However, to further validate its robustness in real-world environments, expanding the dataset to include more diverse and subtle activities will be essential.

In addition, for deployment in safety-critical scenarios, we recommended integrating secondary verification mechanisms, such as CSI-based skeletal modeling or anomaly detection, to verify uncertain patterns and reduce potential risks. These mechanisms can trigger additional validation steps, such as camera activation or caregiver alerts, ensuring reliable operation in sensitive applications.

## Figures and Tables

**Table 1:** Comparison of few-shot learning approaches in Wi-Fi-based HAR.

Method	Predef. Embed. ^1^	New Subj.Adapt. ^2^	Extra Data ^3^	Feature Engr ^4^	General HAR ^5^
ProtoNet	O	X	X	X	O
TOSS	X	O	O	X	O
CSI-GDAM	X	X	O	O	O
CAUTION	X	X	X	O	X
TCN-MAML (Ours)	X	O	X	X	O

^1^ Predef. EMmbed. = requires predefined embeddings. ^2^ New Sunj. Adapt. = adaptable to new subjects. ^3^ Extra Data = needs additional target data. ^4^ Feature Engr. = feature engineering required. ^5^ General HAR = general HAR application.

**Table 2:** TCN-MAML parameters.

Component	Parameter	Value
TCN Backbone	Number of Layers	3
	Dilation Rates	1, 2, 4
	Number of Filters	30, 50, 75
	Kernel Size	15
	Dropout Rate	0.1
Output Shape	Final TCN Output Dimension	$\left(N_{t}\times N_{r},N_{p},N_{f}^{L}\right)$
FCN Classifier	Number of Dense Layers	2
	First Dense Layer Neurons	128
	Second Dense Layer Neurons	64
	Dropout Rate	0.1
	Output Layer Neurons	$12\;(N_{c})$
	Batch Size	32
	Meta-learning Outer Learning Rate	$5\times 10^{-4}$
	Task-level Inner Update Learning Rate	$5\times 10^{-4}$
	Inner-loop Adaptation Steps	5
	Inner-loop Testing Steps	10

**Table 3:** Cross-dataset accuracy comparison.

Dataset	Testing Accuracy (%)
NTU-Fi HAR	99.12
UT-HAR	98.66
HHI	99.6

**Table 4:** A comparison of classification accuracy (%) on the HHI dataset with recent methods.

Method	Accuracy (%)
TCN-AA (Ours)	99.60
SVM	86.21
CSI-IANet	91.30
DCNN	88.66
HHI-AttentionNet	95.47
GraSens	86.00
E2EDLF	86.30
Attention-BiGRU	87.00
H2HI-Net	96.39

**Table 5:** The performances of the proposed augmentation techniques. The augmented dataset contains twice the sample size of the raw dataset.

Augmentation Method	Accuracy (%)			Loss		
	Train	Valid	Test	Train	Valid	Test
Raw data	100	67.2	87.0	0.001	1.098	0.553
Raw + Dropout	99.7	84.2	94.6	0.002	0.184	0.109
Raw + Intra-mixing (30%)	98.8	**86.5**	**94.9**	0.008	**0.178**	**0.055**
Raw + Intra-mixing (20%)	98.2	83.1	90.9	0.01	0.192	0.069
Raw + Inter-mixing (30%)	99.7	77.8	93.3	0.006	0.234	0.082
Raw + Inter-mixing (20%)	98.8	76.8	89.3	0.01	0.223	0.09

Note: Raw data = baseline dataset without augmentation. Dropout = random dropout applied to training samples. Intra-mixing = mixing data within the same class. Inter-mixing = mixing data across different classes.

**Table 6:** The performances of the proposed augmentation techniques. The augmented dataset contains the same sample size as the raw dataset.

Augmentation Method	Accuracy (%)			Loss		
	Train	Valid	Test	Train	Valid	Test
Raw data	100	67.2	87.0	**0.001**	1.098	0.553
(Raw+Dropout)/2	99.1	**67.2**	88.6	0.008	**0.305**	**0.133**
(Raw+Intra-mixing (30%))/2	**100**	66.4	**90.0**	**0.001**	0.361	0.141
(Raw+Intra-mixing (20%))/2	99.2	63.5	87.0	0.006	0.372	0.158
(Raw+Inter-mixing (30%))/2	99.0	64.0	87.6	0.008	0.35	0.172
(Raw+Inter-mixing (20%))/2	95.6	65.0	82.6	0.012	0.363	0.188

Note: Raw data = baseline dataset without augmentation. Dropout = random dropout applied to training samples. Intra-mixing = mixing data within the same class. Inter-mixing = mixing data across different classes.

## Data Availability

The Human-to-Human Interaction (HHI) CSI dataset used in this study is publicly available at https://data.mendeley.com/datasets/3dhn4xnjxw/1. The code for this study is available at https://github.com/Teddy0955/TCN-MAML (both accessed on 5 June 2025).
